# Methylprednisolone Protects Cardiac Pumping Mechanics from Deteriorating in Lipopolysaccharide-Treated Rats

**DOI:** 10.3389/fphys.2015.00348

**Published:** 2015-11-26

**Authors:** Ya-Hui Ko, Ming-Shian Tsai, Ru-Wen Chang, Chun-Yi Chang, Chih-Hsien Wang, Ming-Shiou Wu, Jin-Tung Liang, Kuo-Chu Chang

**Affiliations:** ^1^Department of Physiology, College of Medicine, National Taiwan UniversityTaipei, Taiwan; ^2^Department of Surgery, E-Da HospitalKaohsiung, Taiwan; ^3^Department of Emergency Medicine, National Taiwan University HospitalHsin-Chu, Taiwan; ^4^Department of Surgery, National Taiwan University HospitalTaipei, Taiwan; ^5^Department of Internal Medicine, National Taiwan University HospitalTaipei, Taiwan

**Keywords:** high-mobility group box 1, inflammation, malondialdehyde, maximal systolic elastance, peroxynitrite, theoretical maximum flow

## Abstract

It has been shown that a prolonged low-dose corticosteroid treatment attenuates the severity of inflammation and the intensity and duration of organ system failure. In the present study, we determined whether low-dose methylprednisolone (a synthetic glucocorticoid) can protect male Wistar rats against cardiac pumping defects caused by lipopolysaccharide-induced chronic inflammation. For the induction of chronic inflammation, a slow-release ALZET osmotic pump was subcutaneously implanted to infuse lipopolysaccharide (1 mg kg^−1^ d^−1^) for 2 weeks. The lipopolysaccharide-challenged rats were treated on a daily basis with intraperitoneal injection of methylprednisolone (5 mg kg^−1^ d^−1^) for 2 weeks. Under conditions of anesthesia and open chest, we recorded left ventricular (LV) pressure and ascending aortic flow signals to calculate the maximal systolic elastance (*E*_max_) and the theoretical maximum flow (*Q*_max_), using the elastance-resistance model. Physically, *E*_max_ reflects the contractility of the myocardium as an intact heart, whereas *Q*_max_ has an inverse relationship with the LV internal resistance. Compared with the sham rats, the cardiodynamic condition was characterized by a decline in *E*_max_ associated with the increased *Q*_max_ in the lipopolysaccharide-treated rats. Methylprednisolone therapy increased *E*_max_, which suggests that the drug may have protected the contractile status from deteriorating in the inflamed heart. By contrast, methylprednisolone therapy considerably reduced *Q*_max_, indicating that the drug may have normalized the LV internal resistance. In parallel, the benefits of methylprednisolone on the LV systolic pumping mechanics were associated with the reduced cardiac levels of negative inotropic molecules such as peroxynitrite, malondialdehyde, and high-mobility group box 1 protein. Based on these data, we suggested that low-dose methylprednisolone might prevent lipopolysaccharide-induced decline in cardiac intrinsic contractility and LV internal resistance, possibly through its ability to reduce the aforementioned myocardial depressant substances. However, since our results were obtained in anesthetized open-chest rats, extrapolation to what may occur in conscious intact animals should be done with caution.

## Introduction

Inflammatory disorders such as allergies, asthma, autoimmune diseases, and sepsis are a major cause of illness and death (Cooper and Stroehla, 2003; Riedemann et al., 2003). Lipopolysaccharide (LPS), which is the major bioactive component of the cell membrane of gram-negative bacteria, may promote and amplify inflammatory responses, generating various cardiodepressive mediators (Garner et al., 2003; Carlson et al., 2005; Andreasen et al., 2008). In LPS-induced inflammation, an excessive and prolonged production of nitric oxide (NO) exerts adverse effects on the heart (Kleinert et al., 2004). NO at supraphysiological levels may react with superoxide anions to generate highly toxic compounds such as peroxynitrite to damage cardiac myocytes (Ishida et al., 1996; Khadour et al., 2002). LPS also induces the secretion of high-mobility group box 1 (HMGB1) protein in the myocardium, which may be detrimental to the contractile status of the left ventricle (Li et al., 2006; Xu et al., 2010).

Corticosteroids have been used in the treatment of severe sepsis and septic shock for more than 50 years (Annane et al., 2009). The therapeutic effects of corticosteroids have been characterized by their ability to exert antiinflammatory and immunosuppressive efficacy. Methylprednisolone (MP) is a synthetic glucocorticoid, which can suppress inducible NO synthase (iNOS) expression and other inflammatory molecules through its ability to inhibit nuclear factor-κB (NF-κB) activation (Xu et al., 1998). In 2003, Annane and Cavaillon have shown that a low-dose hydrocortisone can reverse the systemic inflammatory response in subjects secondary to an infection. Moreover, a prolonged low-dose corticosteroid treatment attenuates the severity of inflammation and the intensity and duration of organ system failure (Keh et al., 2003; Oppert et al., 2005). We previously demonstrated that low-dose MP therapy prevents peripheral vasodilation, but stiffens aortas and elastic arteries in rats with LPS-induced chronic inflammation (Ko et al., 2013). Although, MP therapy increases aortic stiffness, the question that remains unanswered is whether LPS-induced impaired cardiac function can be improved by MP therapy associated with the attenuation of cardiosuppressing mediators.

The physical properties of the left ventricle could be reflected in the behavior of the ventricular pressure, volume, and flow (Hunter et al., 1983; Shroff et al., 1983). The relationship between instantaneous ventricular pressure and volume has been considered a time-varying elastance (Sagawa, 1978). The viscouslike behavior can be described as the ventricular pressure-flow relation and can be represented as ventricular internal resistance (Hunter et al., 1983; Shroff et al., 1983; Campbell et al., 1986). For an ejecting beat, the left ventricle as a mechanical pump can be characterized by both the maximal systolic elastance (*E*_max_) and the theoretical maximum flow (*Q*_max_) (Campbell et al., 1986; Shroff et al., 1992). Hunter et al. (1983) showed that *E*_max_ can serve as a reliable index of ventricular contractility because it is independent of preload and afterload, but it is sensitive to changes in the contractile status of the myocardium. Shroff et al. (1992) reported that *Q*_max_ has an inverse relationship with the LV internal resistance, which can be associated with the structural, biochemical, or geometric alterations.

In the present study, we investigated whether low-dose MP therapy can protect male Wistar rats against cardiac pumping defects caused by lipopolysaccharide-induced chronic inflammation. For the induction of chronic inflammation, a slow-release ALZET osmotic pump was subcutaneously implanted to infuse LPS (1 mg kg^−1^ d^−1^) for 2 weeks. The LPS-challenged rats were treated on a daily basis with intraperitoneal injection of MP (5 mg kg^−1^ d^−1^) for 2 weeks. We recorded the LV pressure and ascending aortic flow signals to calculate the *E*_max_ and the *Q*_max_, using the elastance–resistance model (Campbell et al., 1986; Shroff et al., 1992). We also measured myocardial depressant substances such as peroxynitrite and HMGB1 in the LPS-treated rats after MP therapy. Furthermore, the level of cardiac malondialdehyde (MDA) was measured and served as an indirect index of reactive oxygen species (ROS) activity (Gutteridge and Halliwell, 1990).

## Methods

### Animals

We used male Wistar rats, weighing 250–300 g, as experimental animals, which were randomly divided into 4 groups (*n* = 10/group): (i) Sham group, (ii) Sham group treated with MP (Sham-MP), (iii) LPS group, and (iv) LPS group treated with MP (LPS-MP). The range of the applied LPS dosage in rodent was reported from 5 μg kg^−1^ to 5 mg kg^−1^ (Noworyta-Sokołowska et al., 2013). In this study, for low-grade chronic inflammation (Smith et al., 2009), a slow-release ALZET osmotic pump (Model 2004; DURECT Corporation, Cupertino, CA, USA) was subcutaneously implanted to infuse LPS (*Escherichia coli* O55:B5, 1 mg kg^−1^ d^−1^; Sigma–Aldrich, St. Louis, MO, USA) for 2 weeks. The sham group was infused with saline. Both the Sham-MP and the LPS-MP groups were treated on a daily basis with intraperitoneal injection of low-dose MP (5 mg kg^−1^ d^−1^; Pfizer Manufacturing Belgium NV, Puurs, Belgium) for 2 weeks (Ko et al., 2013). The animals were allowed free access to Purina chow and water with a 12-h light–dark cycle. All experiments were conducted according to the *Guide for the Care and Use of Laboratory Animals*. The Animal Care and Use Committee of National Taiwan University approved the study protocol.

### Measurement of plasma levels of free fatty acids (FFA)

Using cardiac puncture technique, blood samples were collected at the end of catheterization. The collected blood samples were subsequently centrifuged at 1600 g at 4°C for 10 min to determine the plasma levels of FFA by using enzymatic kits (Cayman, Ann Arbor, MI, USA and Biovision, Mountain View, CA, USA) (Okabe et al., 1980).

### Proinflammatory cytokine measures

We have previously described procedures to measure proinflammatory cytokines (Ko et al., 2013). In brief, plasma interleukin (IL)-6 (R&D Systems, Minneapolis, MN, USA), C-reactive protein (CRP) (ALPCO, Windham, NH, USA), nitrogen oxides (NO, nitrites + nitrates) (Calbiochem, Merck, Germany), and cardiac peroxynitrite (Cayman Chemical, Ann Arbor, MI, USA) levels were quantified using commercially available enzyme-linked immunosorbent assay kits in strict accordance with the manufacturer's instructions.

### Immunohistochemical staining for inflammatory cells and markers in the myocardium

To examine inflammatory cells and markers in the myocardium, we measured CD68 as macrophage marker and receptor for advanced glycation end products (RAGE) as inflammatory marker.

We have previously described general procedures for immunohistochemical staining technique (Ko et al., 2013). In brief, formalin-fixed rat cardiac tissues were embedded in paraffin, cut into 4-μm-thick sections, and stained with hematoxylin and eosin (H&E). Immunohistocytochemical staining was performed using mouse monoclonal anti-CD68 IgG (MCA341GA) (1:25; AbD Serotec., UK) and goat anti-human RAGE IgG (1:200; AbD Serotec., UK) antibodies in a BenchMark ULTRA slide stainer, and samples were analyzed using an iView Universal DAB Detection kit (Ventana Medical Systems).

### Western blotting for cardiac HMGB1 protein

We have previously described procedures for Western blotting technique (Ko et al., 2013). In brief, Rat cardiac tissue protein samples (50 μg/lane) were resolved using 10% SDS-polyacrylamide gel electrophoresis (SDS-PAGE) and electrotransferred onto polyvinylidene difluoride (PVDF) membranes, which were incubated with a primary monoclonal anti-HMGB1antibody (1:10000; Epitomics, Burlingame, CA, USA) at 4°C overnight, and then incubated with a horseradish peroxidase (HRP)-conjugated secondary immunoglobulin G (IgG) antibody. Immunoreactive bands were visualized using enhanced chemiluminescence (ECL) reagents from PerkinElmer. Relative quantity was obtained by normalizing the density of the target protein against that of β-actin. Experiments were replicated 3 times, and the results are expressed as the mean ± standard error (s.e.).

### Estimation of cardiac MDA level by thiobarbituric acid assay

In this study, thiobarbituric acid reactive substance (TBARS) was used as an estimate of cardiac MDA level, because MDA is not the only physiological molecule that can react with thiobarbituric acid (Del Rio et al., 2005).

We have previously described procedures for measuring MDA/TBARS (Wang et al., 2013). In brief, rat cardiac tissues were stored at −80°C until further analysis. All tissues were homogenized in the RIPA buffer (Sigma Chemical Co., St. Louis, MO, USA) with a 1% protease inhibitor cocktail (Sigma Chemical Co.) and centrifuged at 1600 g at 4°C for 10 min to obtain supernatants for measuring cardiac MDA level. LV MDA levels were estimated by TBARS by using a commercial kit (Cayman Chemical Company, Ann Arbor, MI, USA) (Beuge and Aust, 1978). Protein concentrations of the left ventricle were assayed using the Bradford method (DCProtein Assay; Bio-Rad) (Bradford, 1976).

### Catheterization

General surgical procedures and measurement of cardiodynamic variables have previously been described in anesthetized rats (Chang et al., 2002). In brief, the rats were anesthetized with sodium pentobarbital (50 mg kg^−1^, i.p.), placed on a heating pad, and intubated and ventilated with a Model 131 rodent respirator (New England Medical Instruments, Medway, MA, USA). The rectal temperature of each rat was measured by using clinical thermometer. The chest was opened through the second intercostal space of the right side. The pulsatile aortic flow was recorded by using an electromagnetic flow probe, Model 100 series, with an internal circumference of 8 mm (Carolina Medical Electronics, King, NC, USA), which was positioned around the ascending aorta. The LV pressure was measured by using a high-fidelity pressure catheter, Model SPC 320 of size 2F (Millar Instruments, Houston, TX, USA), which was inserted through the isolated right carotid artery into the left ventricle. An ECG/Biotach amplifier (Gould, Cleveland, OH, USA) was used to record lead II electrocardiogram (ECG). Using the peak R wave of ECG as a fiducial point, we selected the LV pressure and aortic flow signals of 5–10 beats and averaged these signals in the time domain. To characterize the pumping mechanics of an inflamed heart, a single-beat estimation technique was performed to generate model parameters for calculating the systolic elastance and resistance (Chang and Kuo, 1997; Chang, 1998).

### Prediction of the LV pressure by using the elastance–resistance model

We have previously described procedures for characterizing the cardiac pumping mechanics from the elastance-resistance model (Wu et al., 2008; Wang et al., 2013). In brief, one can predicted the model-derived pressure of the left ventricle $\overset{\wedge }{P}\left(t\right)$ if the model parameters could be previously identified (Campbell et al., 1986; Shroff et al., 1992). The following equation describes the relationship between the instantaneous LV pressure, isovolumic pressure, and aortic flow:

(1) $$\begin{array}{c} \overset{\wedge }{P}(t)=P_{iso}(t)\left[1-\frac{V_{ej}(t)}{V_{eed}}\right]\left[1-\frac{Q(t)}{Q_{\max}}\right] \end{array}$$

*V*_*ej*_(*t*) is the instantaneous ejected volume, which was computed by numerically calculating the running integral of the aortic flow signal *Q*(*t*). *Q*_max_ is the theoretical maximum flow, *V*_*eed*_ is the effective LV end-diastolic volume that is the volume difference between the LV end-diastolic volume and the zero-pressure volume axis intercept, and *P*_*iso*_(*t*) is the isovolumic pressure obtained by occluding the ascending aorta near the sinuses of Valsalva at the end of diastole. In this study, *P*_*iso*_(*t*) was derived from the measured pressure of an ejection contraction by using a nonlinear least-squares approximation technique, as follows (Sunagawa et al., 1980):

(2) $$P_{iso}(t)=\frac{1}{2}P_{id\max}\left[1-\cos(\omega t+c)]+P_{d}\right]$$

**Figure 1:**
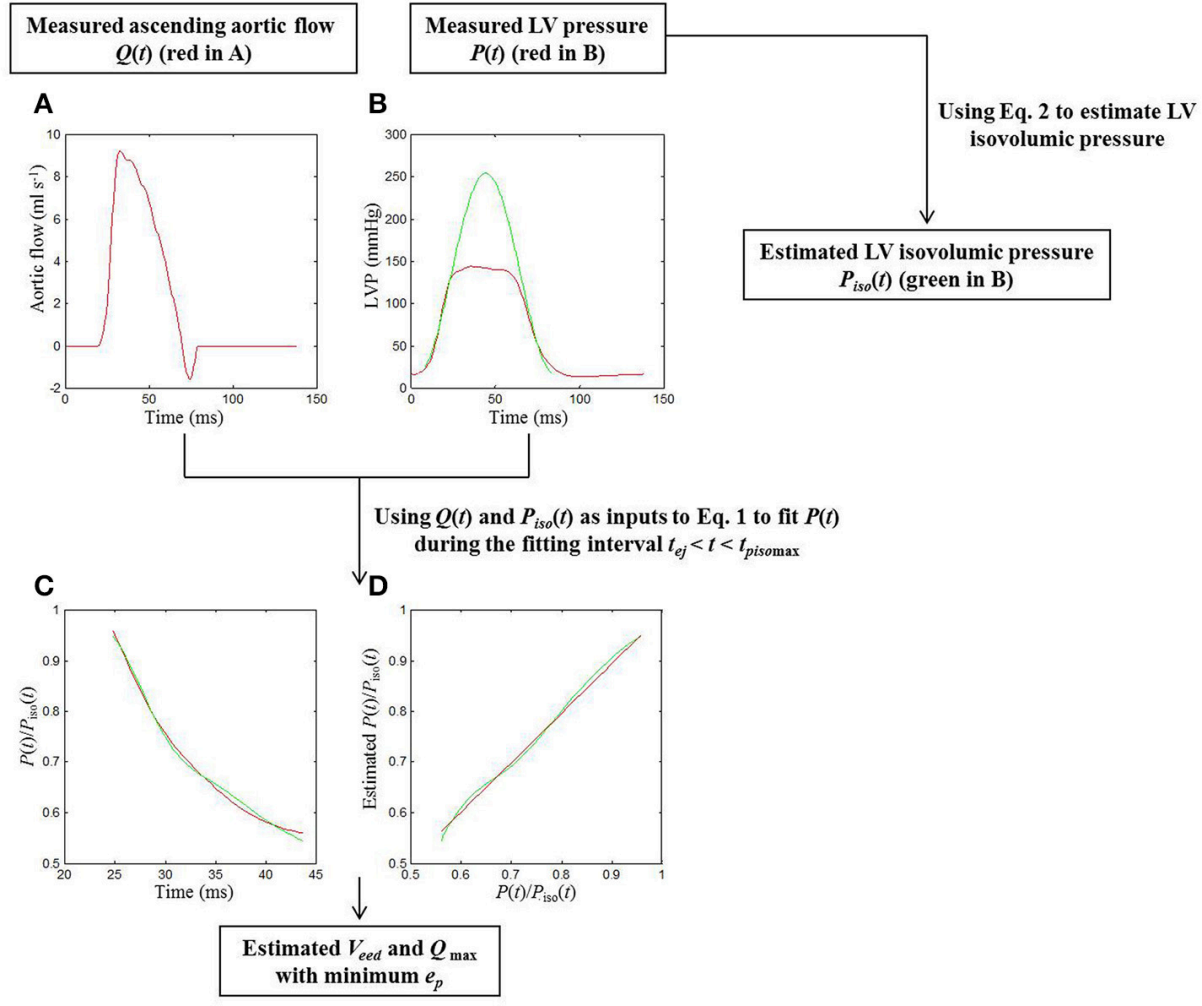
**Procedures for the estimation of cardiodynamic parameters using the elastance-resistance model in a sham rat**. In Graph **(A)**, the red curve shows the measured *Q(t)*. In Graph **(B)**, the isovolumic portions of the measured *P*(*t*) (the red curve) were used to estimate the *P*_*iso*_(*t*) (the green line) using (Equation 1). Taking *Q*(*t*) and *P*_*iso*_(*t*) as 2 inputs into (Equation 2), a good similarity between the computed and measured *P*(*t*) was observed when the (Equation 2) was fit over *t*_*ej*_ < *t* < *t*_*piso*max_, as shown in **(C,D)**. In Graph **(C)**, the measured data are described in terms of the red line; the model-derived data are represented by the green line. In Graph **(D)**, the scatter diagram between measured and model-generated data is described by using the green line. The red line is the simple linear regression line of measured and model-generated data, with a slope of approximately 1 (0.973). Thus, the model parameters *V*_*eed*_ and *Q*_max_ were determined with the minimum *e*_*p*_. *e*_*p*_, root-mean-square error; LV, left ventricular; *P*(*t*), measured LV pressure; *P*_*iso*_(*t*), estimated LV isovolumic pressure; *P*(*t*)/*P*_*iso*_(*t*), ratio of *P*(*t*) to *P*_*iso*_(*t*); *Q*(*t*), measured ascending aortic flow; *Q*_max_, theoretical maximum flow; *t*_*ej*_, the onset of ventricular ejection; *t*_*piso*max_, the time of the peak isovolumic pressure; *V*_*eed*_, effective LV end-diastolic volume.

*P*_*id*max_ is the peak-developed isovolumic pressure, ω is the angular frequency, *c* is the phase-shift angle of the sinusoidal curve, and *P*_*d*_ is the LV end-diastolic pressure. The isovolumic pressure *P*_*iso*_(*t*) (green curve in Figure 1B) was obtained by fitting the measured LV pressure curve segments from the end-diastolic pressure point to the peak +*dP*/*dt* and from the pressure point of the peak –*dP*/*dt* to the same level as the end-diastolic pressure of the preceding beat (Takeuchi et al., 1991). The LV end-diastolic point was identified using the peak of the ECG R wave. The estimated peak isovolumic pressure *P*_*iso*max_is the pressure sum of *P*_*id*max_ and *P*_*d*_.

Both *V*_*eed*_ and *Q*_max_ in Equation (1) are the model parameters remained to be determined. Campbell et al. (1986) demonstrated that Equation (1) can be used to fit the measured LV pressure of an ejecting beat precisely, if the fitting interval is *t*_*ej*_ < *t* < *t*_*piso*max_, where *t*_*ej*_ is the onset of ventricular ejection and *t*_*piso*max_is the time of peak isovolumic pressure. The Nelder–Meade simplex algorithm was used in a nonlinear least-squares manner to iteratively adjust *V*_*eed*_ and *Q*_max_ to minimize the root-mean-square error (*e*_*p*_) (Dennis and Woods, 1987). The parameters coincident with the minimum *e*_*p*_ were recorded as the model estimates of the LV systolic pumping mechanics (green curves in Figures 1C,D). Thus, the LV systolic elastance was calculated using *E*(*t*) = *P*_*iso*_(*t*)/*V*_*eed*_, and its maximal value is the maximal systolic elastance (*E*_max_ = *P*_*iso*max_/*V*_*eed*_). The internal resistance of the left ventricle was expressed as *R*(*P*_*iso*_) = *P*_*iso*_(*t*)/*Q*_max_ (Shroff et al., 1992).

### LV end-systolic equilibrium point

**Figure 2:**
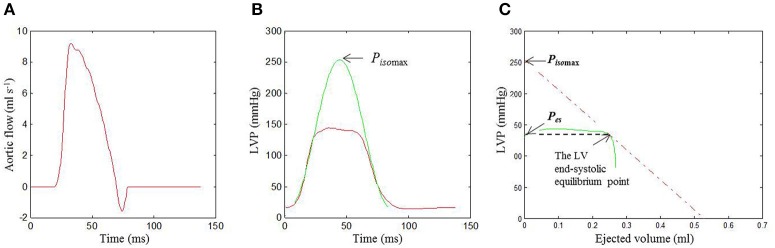
**Identification of the LV end-systolic equilibrium point in the same rat as shown in Figure 1**. The time integration of *Q*(*t*) **(A)** and the measured *P*(*t*) (red curve in **B**) were used to construct the pressure-ejected volume loop (green curve in **C**). In Graph **(B)**, the isovolumic portions of the measured *P*(*t*) were used to estimate the *P*_*iso*_(*t*) (the green line) using (Equation 1). In **(C)**, drawing a tangential line from *P*_*iso*max_ to the right corner of the pressure-ejected volume loop yields a point referred to as the end-systolic equilibrium point. LV, left ventricular; *P*(*t*), measured LV pressure; *P*_*iso*_(*t*), estimated LV isovolumic pressure; *P*_*iso*max_, estimated peak LV isovolumic pressure; *Q*(*t*), measured ascending aortic flow.

We have previously described procedures for defining the LV end-systolic equilibrium point (Wang et al., 2013). In brief, the time integration of aortic flow (red curve in Figure 2A) and the measured LV pressure (red curve in Figure 2B) were used to construct the pressure-ejected volume loop. After drawing a tangential line from the estimated *P*_*iso*max_ to the right corner of the pressure-ejected volume loop, we then obtained a point referred to as the end-systolic equilibrium point (Barnea and Jaron, 1990; Chang, 1998), as shown in Figure 2C. The pressure at this equilibrium point is the LV end-systolic pressure (*P*_*es*_).

### Statistical analysis

Results are expressed as the mean ± s.e. Two-way ANOVA was used to determine the statistical significance, when multiple comparisons were made among the effects of LPS and MP on cardiodynamic and biochemical data. Significant differences were assumed at the level of *P* < 0.05. If the ANOVA results for any cardiodynamic or biochemical variable reached the level of significance, then Tukey's honestly significant difference method was used to determine which groups of rats had different mean values of that variable.

## Results

Compared with the age-matched shams, there were no significant differences in the body weight (BW), whole heart weight, LV weight (LVW), and LVW/BW ratio in the LPS-challenged animals, as shown in Table 1. Although, MP therapy markedly reduced heart weight, BW and LVW, it did not alter the LVW/BW ratio in the LPS-treated rats. By contrast, only BW was decreased by MP in the Sham group. Neither LPS nor MP affected *HR*. By contrast, an increase in *CO* associated with the decreased *P*_*es*_ was observed in the LPS-treated rats. However, these 2 parameters did not respond to MP therapy. MP therapy for 2 weeks had improved *dP*/*dt*_max_ and *dP*/*dt*_*min*_, which were impaired by LPS challenge. Those hemodynamic variables were not affected by MP in the Sham-MP group.

**Table 1:** **Basic and hemodynamic data in the LPS-challenged rats treated with MP**.

	**Sham (*n* = 10)**	**Sham-MP (*n* = 10)**	**LPS (*n* = 10)**	**LPS-MP (*n* = 10)**
BW	355.0±9.8	332.3±13.8^†^	376.2±9.3	318.8±7.2^‡^
Heart weight	1.01±0.03	0.94±0.05	1.07±0.04	0.89±0.04^‡^
LVW	0.66±0.02	0.65±0.03	0.69±0.03	0.61±0.02^‡^
LVW/BW	1.85±0.05	1.89±0.08	1.89±0.07	1.91±0.06
RT	36.2±0.2	36.4±0.1	36.6±0.2	36.5±0.2
HR	424.8±10.5	423.7±11.8	424.1±13.0	443.4±10.2
CO	1.943±0.132	2.170±0.133	2.541±0.094^†^	2.456±0.095
*P*_*es*_	125.5±4.9	108.7±2.8	102.2±4.2^†^	111.9±4.5
*dP/dt*_max_	10503.9±503.6	10460.7±417.2	8720.9±438.9^†^	10847.1±630.5^‡^
*dP/dt*_*min*_	−7605.7±265.1	−7011.7±225.5	−6121.4±311.3^†^	−7726.2±302.7^‡^

*All values are expressed as the mean ± s.e. BW, body weight (g); CO, cardiac output (mL s^−1^); dP/dt_max_ (mmHg s^−1^); dP/dt_min_ (mmHg s^−1^); HR, basal heart rate (beats min^−1^); LVW, left ventricular weight (g); LVW/BW, LVW to BW ratio (mg/g); P_es_, LV end-systolic pressure (mmHg); RT, rectal temperature (°C); LPS, lipopolysaccharide; MP, methylprednisolone; LPS-MP, LPS rats treated with MP; Sham-MP, Sham treated with MP*.

† *P < 0.05 from the sham group*.

‡ *P < 0.05 from the LPS group*.

As for the inflammatory cytokines, MP therapy attenuated the LPS-induced increase in the plasma CRP, IL-6, and NO levels, as shown in Table 2. By contrast, the plasma FFA levels were elevated by treating both the sham and LPS rats with MP.

**Table 2:** **Proinflammatory cytokines and plasma FFA in the LPS-challenged rats treated with MP**.

	**Sham (*n* = 10)**	**Sham-MP (*n* = 10)**	**LPS (*n* = 10)**	**LPS-MP (*n* = 10)**
CRP	256.4 ± 13.0	268.0±14.4	359.0 ± 19.3^†^	307.3 ± 16.1^‡^
IL-6	25.8 ± 1.8	27.3 ± 2.1	53.7 ± 5.9^†^	32.3 ± 4.5^‡^
NO	16.1 ± 1.8	17.9 ± 1.1	35.3 ± 1.8^†^	21.4 ± 1.6^‡^
FFA	0.88 ± 0.01	0.93 ± 0.02^†^	0.86 ± 0.05	0.93 ± 0.10^‡^

*All values are expressed as the mean ± s.e. CRP, plasma C-reactive protein (mg mL^−1^); FFA, free fatty acid (mM); IL-6, plasma interleukin-6 (pg mL^−1^); NO, plasma nitrites and nitrates (μM); LPS, lipopolysaccharide; MP, methylprednisolone; LPS-MP, LPS rats treated with MP; Sham-MP, Sham treated with MP*.

† *P < 0.05 from the sham group*.

‡ *P < 0.05 from the LPS group*.

The red curves in Figures 1A,B show the measured ascending aortic flow signal and LV pressure waveform, respectively, in a sham rat. The green line in Figure 1B represents the isovolumic pressure–time curve at the measured end-diastolic volume, which was estimated by fitting a sinusoidal function to the isovolumic portions of the measured LV pressure. Similarity between the computed and measured LV pressure waveforms during the fitting interval *t*_*ej*_ < *t* < *t*_*piso*max_ was shown in Figures 1C,D. The parameter *e*_*p*_ was used as an indicator of the quality of fit, and its averaged value for all animals (*n* = 40) studied was 0.0036 ± 0.0002. A high coefficient of determination (0.9916 ± 0.0006) and a relatively low standard error of the estimate (2.11 ± 0.11%) reflected the goodness of fit of the elastance-resistance model. All these data indicated that the model-generated parameters *V*_*eed*_ and *Q*_max_ were acceptable for the analysis of cardiac pumping mechanics.

Figure 2C demonstrates the end-systolic equilibrium point, which was determined by drawing a tangential line from *P*_*iso*max_ to the right corner of the pressure-ejected volume loop (green curve).

**Figure 3:**
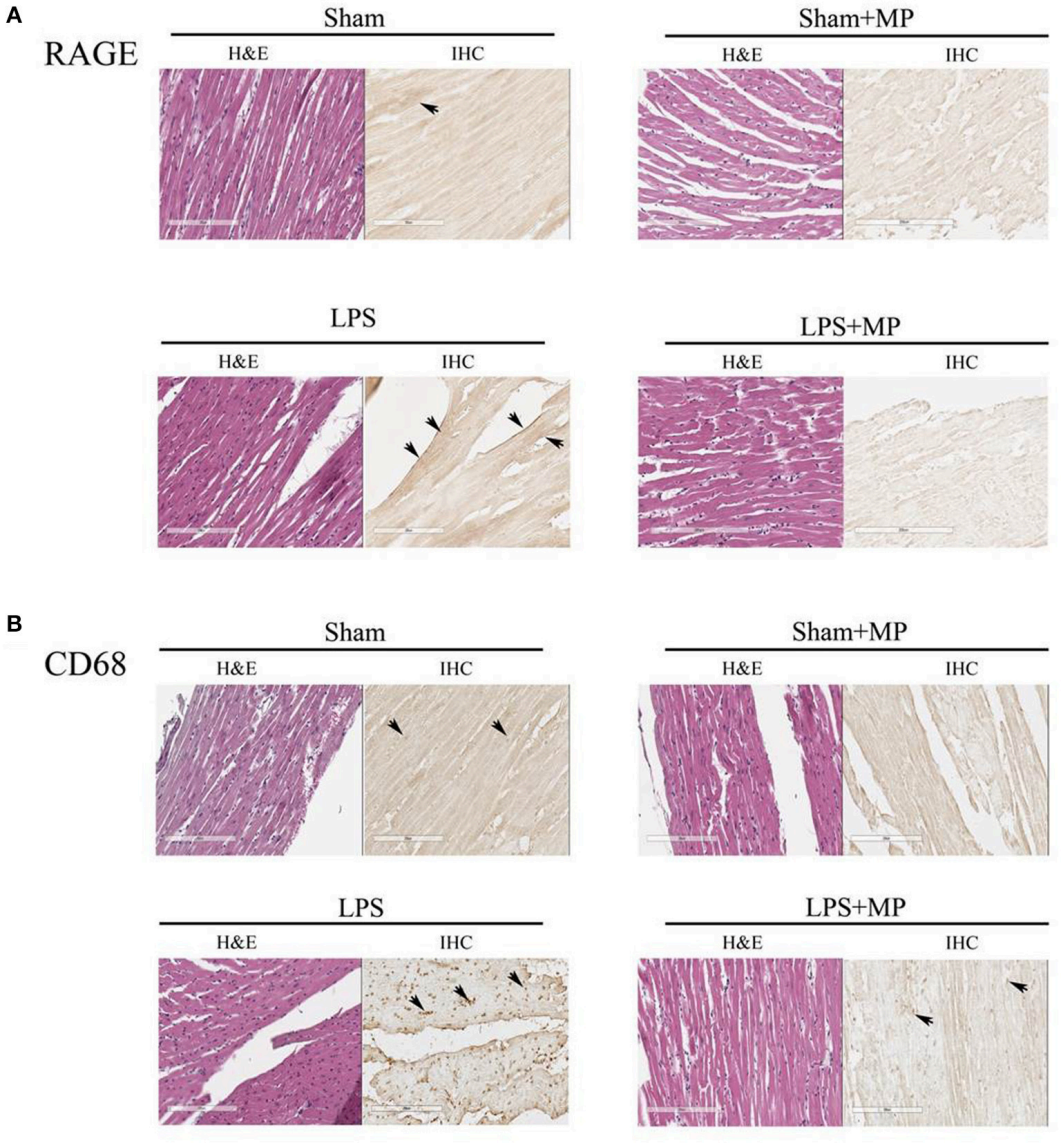
**Effects of LPS and MP on the expression of CD68 (macrophage marker) (A) and RAGE (inflammatory marker) (B) in the myocardium**. We used the immunohistochemical staining technique to probe CD68 (400x) and RAGE (400x) expressions at the cardiac tissues. The sites of antibody staining were indicated by arrows. LPS, lipopolysaccharide; MP, methylprednisolone; RAGE, receptor for advanced glycation end products.

Using the immunohistochemical staining technique, we demonstrated that the LPS-challenged rats displayed greater immunoreactivity of RAGE (Figure 3A) and CD68 (Figure 3B) proteins than did the aged-matched shams. Figure 3 also shows that MP therapy prevented the LPS-induced increase in CD68 and RAGE expressions in the rat myocardium. The immunoreactivities of CD68 and RAGE proteins for each group were compared with their untreated H&E stains in Figure 3.

**Figure 4:**
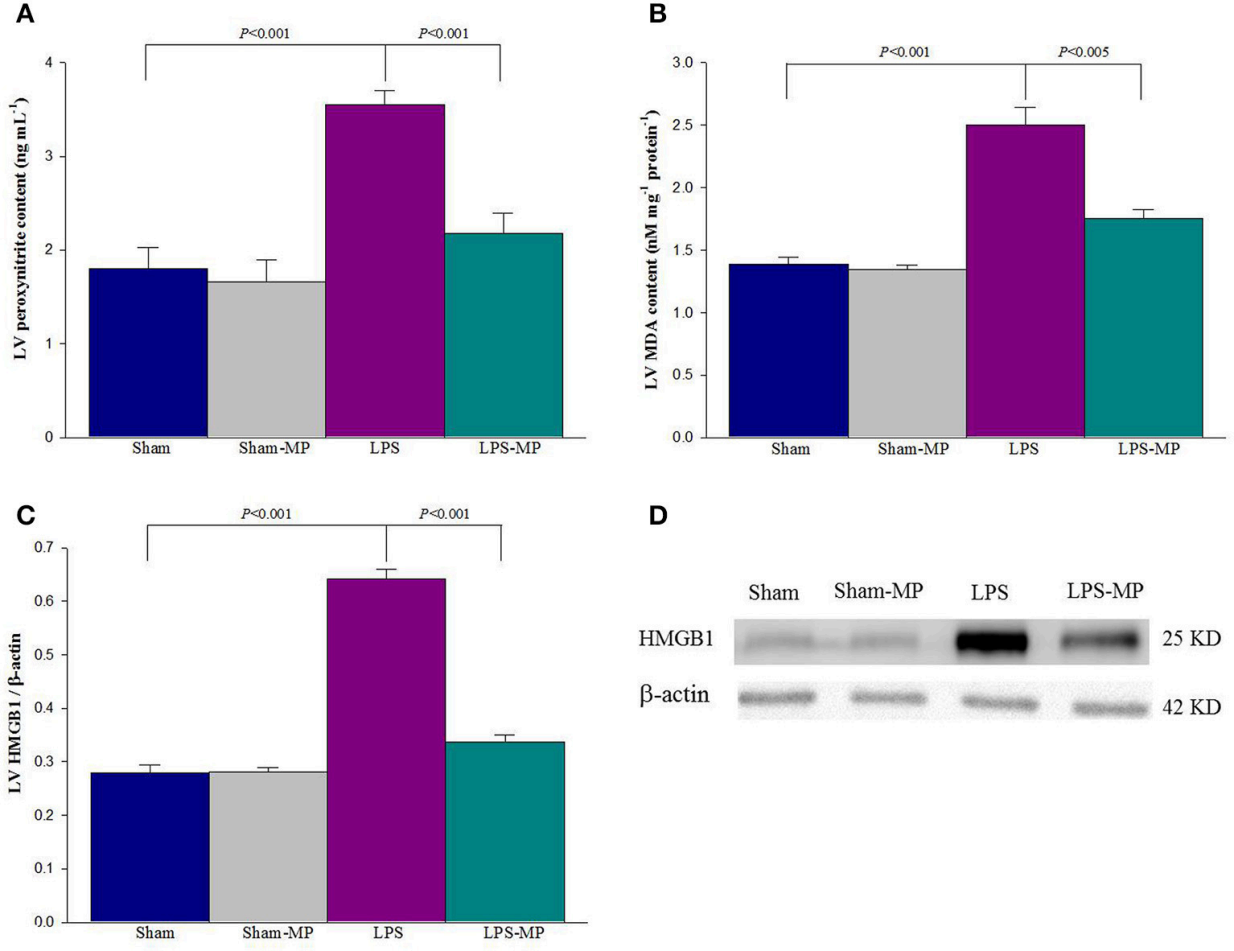
**Effects of LPS and MP on cardiac peroxynitrite (A), MDA (B), and HMGB1 (C) levels**. Graph **(D)** shows the SDS-PAGE profiles of LV HMGB1, which were electrotransferred onto PVDF membranes by using an anti-HMGB1 antibody. Using β-actin expression as a referred base, the expression of protein was normalized to calculate the LV HMGB1 content. Cardiac peroxynitrite level was measured using commercially available enzyme-linked immunosorbent assay kits. LV MDA levels were estimated by TBARS by using a commercial kit. Results are expressed as the mean ± s.e. LPS, lipopolysaccharide; LV, left ventricular; MP, methylprednisolone; HMGB1, high mobility group box 1; MDA, malondialdehyde (*n* = 10/group).

Figure 4 shows the effects of MP and LPS on the expression of myocardial depressants such as peroxynitrite (A), MDA (B), and HMGB1 (C) in the rats studied. The SDS-PAGE profiles of the LV HMGB1 protein is shown in Figure 4D after we performed Western blotting. Cardiac levels of the aforementioned depressive mediators were higher in the LPS-treated rats than in the aged-matched shams. MP therapy prevented the LPS-induced increase in cardiac HMGB1, peroxynitrite, and MDA levels. Neither HMGB1 nor peroxynitrite and MDA were attenuated by MP in the Sham group.

**Figure 5:**
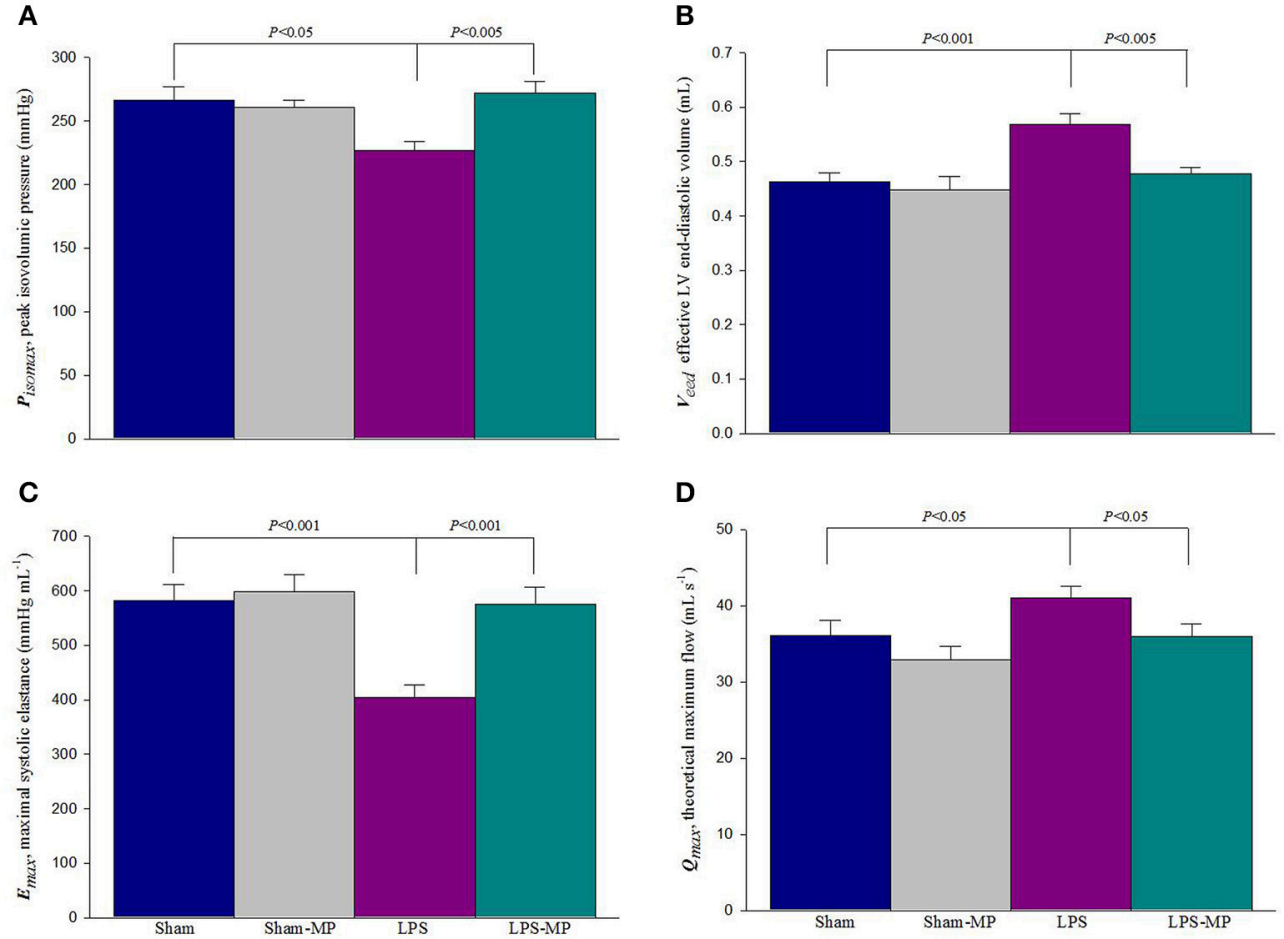
**Effects of LPS and MP on ***P***_***iso***max_ (A), ***V***_***eed***_ (B), ***E***_max_ (C), and ***Q***_max_ (D)**. The ratio of *P*_*iso*max_ to *V*_*eed*_ determines the LV *E*_max_. Results are expressed as the mean ± s.e. LPS, lipopolysaccharide; LV, left ventricular; MP, methylprednisolone; *P*_*iso*max_, estimated peak isovolumic pressure; *V*_*eed*_, effective LV end-diastolic volume; *E*_max_, maximal systolic elastance; *Q*_max_, theoretical maximum flow (*n* = 10/group).

As a mechanical pump, 2 independent features of the left ventricle can be described using the systolic elastance and internal resistance. Figure 5 shows the effects of MP and LPS on the estimated *P*_*iso*max_ (A), *V*_*eed*_ (B), *E*_max_ (C), and *Q*_max_ (D). LPS challenge reduced *P*_*iso*max_ and significantly increased *V*_*eed*_, resulting in a marked decrease in *E*_max_. After MP therapy, the LPS-treated rats significantly reduced *V*_*eed*_ and significantly increased *P*_*iso*max_ and *E*_max_. When normalized for LVW, *E*_max_ of the LPS-MP heart (i.e., *E*_max*n*_ = *E*_max_/LVW) was still significantly higher than that of the LPS heart (972.9 ± 75.2 vs. 587.9 ± 33.8 mmHg mL^−1^ g^−1^, *P* < 0.001). MP therapy prevented an LPS-induced increase in *Q*_max_. Neither *E*_max_ nor *Q*_max_ was influenced by MP administered to the Sham group.

**Figure 6:**
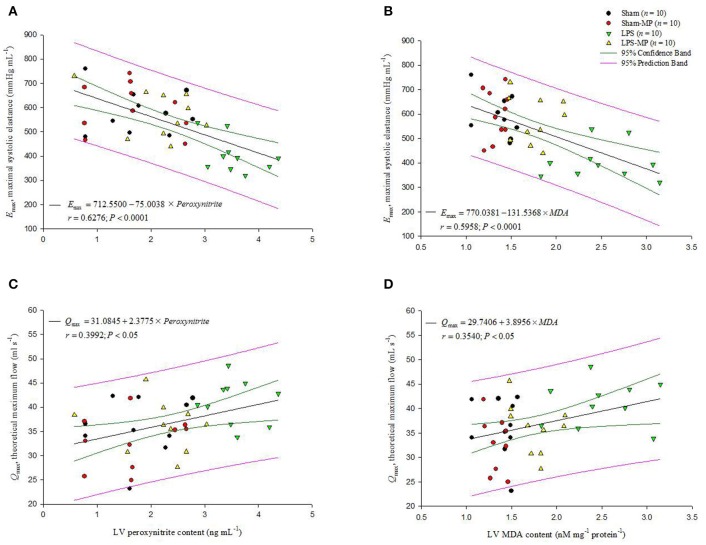
**Association of cardiodynamic parameters with the cardiac depressant substances**. At the cardiac tissues, the LV *E*_max_ is inversely related with either LV peroxynitrite level **(A)** or LV MDA content **(B)**. By contrast, the LV *Q*_max_ increased in response to an increase in LV peroxynitrite level **(C)** or to the increased LV MDA content **(D)**. *E*_max_, maximal systolic elastance; *Q*_max_, theoretical maximum flow; MDA, malondialdehyde (*n* = 10/group).

The association of cardiodynamics and cardiac depressant substances was shown in Figure 6. At the cardiac tissues, LV *E*_max_ was significantly diminished by the increased peroxynitrite (*r* = 0.6276, *P* < 0.0001 in A) as well as MDA contents (*r* = 0.5958, *P* < 0.0001 in B), respectively. By contrast, as LV peroxynitrie level was increased by LPS challenge, the rats had an augmentation in LV *Q*_max_ (*r* = 0.3992, *P* < 0.05 in C). The LV *Q*_max_ was also in a positive linear relationship with the LV MDA content (*r* = 0.3540, *P* < 0.05 in D).

## Discussion

For MP therapy, the range of the applied dosage in rodent is from 1 mg kg^−1^ to 40 mg kg^−1^ (Ghellioni et al., 2015; Ren et al., 2015). In 2010, Guess et al. have suggested that short-term, high-dose glucocorticoid treatment resulted in similar changes in gene expression and glucocorticoid receptor phosphorylation to that of long-term, low-dose glucocorticoid treatment (Guess et al., 2010). Moreover, Annane and Cavaillon (2003) have shown that a low-dose (but not a high-dose) hydrocortisone can reverse the systemic inflammatory response, endothelial activation, and coagulation disorders in subjects secondary to an infection. At these low doses, prolonged corticosteroid therapy has the potential to improve rather than to suppress innate immunity in patients with septic shock (Kaufmann et al., 2008). In the present study, the low-dose MP therapy protected the LPS-challenged rats from deterioration in cardiac pumping mechanics associated with the reduced cardiac levels of negative inotropic molecules such as peroxynitrite, MDA, and HMGB1 protein. Our study results were consistent with the findings of other studies (Keh et al., 2003; Oppert et al., 2005); prolonged low-dose corticosteroid treatment attenuates the severity of inflammation and the intensity and duration of organ system failure.

As mentioned, the ratio of *P*_*iso*max_ to *V*_*eed*_ determines the LV systolic elastance (*E*_max_). In this study, the reduced *E*_max_ in inflammatory heart disease (Figure 5C) was evidenced by the fact that *P*_*iso*max_ was significant decreased by 14.9% (Figure 5A) and *V*_*eed*_ increased 23.2% (Figure 5B) in rats after LPS challenge. The diminished cardiac contractility of the inflamed heart indicated that the myocardium of the LPS-treated rats could not produce sufficient pressure to support *E*_max_ along with the increased *V*_*eed*_. After MP therapy for 2 weeks, the LPS-associated changes in both *P*_*iso*max_ and *V*_*eed*_ improved the cardiac contractile status, as shown by a 42.4% increase in *E*_max_. Another feature of cardiac pumping mechanics that was altered in the LPS-treated rats was the decreased LV internal resistance, as shown by the increase in *Q*_max_ (Figure 5D). After MP therapy, a decline in *Q*_max_ indicated that the LV internal resistance of the inflamed hearts was restored to normal in the LPS-treated rats. In parallel, the benefits of MP on the LV systolic pumping mechanics were associated with its ability to reduce the cardiac levels of peroxynitrite, malondialdehyde, and HMGB1 molecules in inflammation (Figure 4).

We previously demonstrated that rats challenged with LPS had concomitant enhancement of advanced glycation end products (AGEs), RAGE, and iNOS expressions within the vessel wall (Ko et al., 2013). The interaction of AGEs with its receptor RAGE maintained and even amplified inflammatory activities. In the present study, long-term LPS challenge exerted inflammation on cardiac tissues, as evidenced by the increased CD68 and RAGE expressions in the heart (Figure 3). An LPS challenge also enhanced and prolonged the release of CRP, IL-6, and NO in the plasma (Table 1). In cardiac tissues, the overproduction of myocardial peroxynitrite (Figure 4A) in the LPS-treated rats might be one of the factors responsible for the impaired pumping mechanics of the inflamed heart. We found that the LV *E*_max_ was significantly inversely associated with the LV peroxynitrite (Figure 6A), whereas the LV *Q*_max_ was enhanced by the increased LV peroxynitrite (Figure 6C). This result was supported by that of the study by Gao et al. (2003) in which the generation of myocardial peroxynitrite caused cytokine-induced myocardial contractile dysfunction. MP therapy for 2 weeks reduced cardiac peroxynitrite levels in the LPS-treated rats (Figure 4A), restoring the cardiac contractility (Figure 6A) and LV internal resistance (Figure 6C) to normal. This MP-induced decline in myocardial levels of peroxynitrite might partially explain the prevention of LPS-induced injury in the systolic pumping mechanics of the left ventricle.

LPS stimulation also resulted in concomitant enhancement of myocardial depressant substances, such as MDA (Figure 4B) and HMGB1 (Figure 4C), at the cardiac level. MDA is a highly toxic substance, which is generated by lipid peroxidation (Slatter et al., 2000). MDA serves as an indirect index of ROS activity (Gutteridge and Halliwell, 1990) because of its ability to significantly increase oxidative stress and superoxide production (Shanmugam et al., 2008). Membrane damage caused by ROS-induced lipid peroxidation has been considered the predominant mechanism for cellular membrane dysfunction and subsequent alteration of cellular functions (Porter et al., 1995; Dhalla et al., 1996). Thus, the increase in cardiac MDA levels (Figure 4B) caused by an LPS challenge might play a negative role in the regulation of cardiac pumping mechanics. We found that the LV *E*_max_ deteriorated with the increased LV MDA content (Figure 6B). By contrast, the LV *Q*_max_ was augmented by the increased LV MDA molecule (Figure 6D). This result supports that of the study by Folden et al. (2003), in which MDA directly induced cardiac depression because of its ability to enhance oxidative stress at the ventricular myocyte level. The LPS-induced increase in myocardial levels of MDA was attenuated by MP therapy (Figure 4B), and the ability of MP to reduce cardiac MDA levels might allow the drug to improve the systolic mechanical behavior of the ventricular pump in the LPS-treated rats (Figures 6B,D).

Another factor responsible for cardiac contractile dysfunction associated with endotoxemia is HMGB1 (Hagiwara et al., 2008), a damage-associated molecular pattern molecule that is actively secreted by stimulated immune cells and passively released by nonimmune cells undergoing necrosis (Klune et al., 2008). In the present study, observed was the association of the myocardial depressant HMGB1 molecule with the deterioration of cardiac pumping mechanics regarding *E*_max_ and *Q*_max_ in rats after an LPS challenge (Figure 4C). This result was supported by that of the study by Xu et al. (2010), in which LPS challenge induced HMGB1 secretion by viable cardiac myocytes, and HMGB1 affected the LPS-induced myocardial contractile dysfunction. The LPS-induced depression of cardiac pumping mechanics was prevented by MP therapy, which might be associated with the attenuation of LPS-induced cardiomyocyte production and HMGB1 secretion in intact animals.

Although, no mechanisms were provided in this study to explain how MP reduced those cardiac depressant substances, Bains and Hall (2012) demonstrated that MP has the potential to inhibit peroxynitrite and lipid peroxidation-derived MDA content. The mode of the therapeutic action of glucocorticoids involves an inhibition of LPS-induced HMGB1 release, which is mediated by calcium-dependent classical PKC (Oh et al., 2009). Our study results offer a basic consideration for the clinical studies exploring the benefits of prolonged low-dose corticosteroid treatment on cardiac failure in patients with inflammatory disorder.

Several investigators found that treating rats with MP resulted in a reduction in BW and an increase in plasma glucose, insulin, and FFA concentrations (Skjaerbaek et al., 1999; Fang et al., 2011). Glucose and insulin feedback interactions were extended to capture the major metabolic effects of FFA in stimulation of insulin secretion and inhibition of insulin-stimulated glucose utilization (You et al., 2009). Thus, MP may cause catabolism to deteriorate insulin action, lowering glucose utilization. As a result, the body cannot synthesize proteins, ultimately leading to weight loss. In the present study, we found that there existed BW loss and higher plasma FFA in both the Sham-MP and LPS-MP groups. Moreover, no weakness and abnormal death were observed during the experiment study.

In this study, we provided an approach to consider the clinical application of an elastance–resistance model in the study of cardiodynamics. In clinical setting, the indispensable isovolumic signals obtained by occluding the ascending aorta at the end of diastole are not permitted in human subjects. This critical problem could be solved by using a curve-fitting technique to obtain the isovolumic pressure curve from the instantaneous pressure of an ejecting contraction (Sunagawa et al., 1980). With the estimated isovolumic pressure, the elastance–resistance model showed a satisfactory quality of the model fit when evaluating the ventricular elastance and resistance. Moreover, without any perturbation of the loading conditions, the single-beat estimation technique allowed investigators to compute the 2 aforementioned LV systolic parameters from the pulsatile LV pressure and ascending aortic flow signals, which were obtained over a single cardiac cycle.

Certain limitations of this study deserve consideration. Because both the pulsatile LV pressure and ascending aortic flow signals cannot be measured in conscious animals, our study results pertain only to measurements made in the anesthetized open-chest rat. A fall in blood pressure may occur in this experimental setting, introducing reflex effects not found in the close-chest setting (Zuckerman and Yin, 1989). To what extent the effects of anesthesia and thoracotomy are on pulsatile cardiac dynamics in the rat remains uncertain. Thus, it should be emphasized that heart function evaluated under conditions of anesthesia and open chest is a limitation of this study. This limitation may also apply to other measurements, such as blood parameters and histological assessments. Extrapolation of our observations to intact conscious animals should be done with caution.

Another disadvantage of this study is that no cardiac echo examination was performed to compare the functional phenotype of the heart with the intrinsic activity of the myocardium obtained by using the elastance-resistance model analysis. However, in addition to cardiac intrinsic contractility expressed as *E*_max_, cardiac disease can significantly affect ventricular resistance in terms of *Q*_max_ (Shroff et al., 1983). Although, there is a practical advantage to perform echography or telemetry, it is difficult to evaluate ventricular resistance based on this kind of measurement.

In summary, a concomitant increase in cardiac peroxynitrite, MDA, and HMGB1 levels was observed in rats with a long-term LPS challenge. These myocardial depressants might cause adverse effects on the systolic mechanical behavior of the ventricular pump regarding *E*_max_ and *Q*_max_. After low-dose MP therapy for 2 weeks, the elevated cardiac levels of peroxynitrite, malondialdehyde, and HMGB1 molecules were attenuated in rats with LPS-induced inflammation. In parallel, MP therapy ameliorated the contractile dysfunction of the left ventricle in LPS-treated rats, as evidenced by the increase in *E*_max_. Moreover, administering MP therapy to the LPS-treated animals restored the reduced LV internal resistance of the inflamed heart to normal, as shown by the decrease in *Q*_max_. All these findings suggest that the benefits of low-dose MP on cardiac pumping mechanics might be associated with its ability to reduce the aforementioned cardiodepressive molecules in rats with LPS-induced chronic inflammation.

## Author contributions

YK and MT developed concept and designed study. YK and RC performed animal experiment. YK and KC wrote manuscript. CC and MW collected data, and performed statistical analysis. MT and CW provided advice on surgical procedure. JL provided great advice on revision of the paper. KC supervised this work. All authors read and approved the final manuscript.

### Conflict of interest statement

The authors declare that the research was conducted in the absence of any commercial or financial relationships that could be construed as a potential conflict of interest.

## Abbreviations

*CO*: cardiac output
CRP: C-reactive protein
*E*_max_: maximal systolic elastance
HMGB1: high-mobility group box 1
*HR*: basal heart rate
IL-6: interleukin-6
LPS: lipopolysaccharide
LV: left ventricular
MDA: malondialdehyde
MP: methylprednisolone
NO: nitric oxide
*Q*_max_: theoretical maximum flow
*P*_*es*_: LV end-systolic pressure
*P*_*iso*_: LV isovolumic pressure
*P*_*iso*max_: peak LV isovolumic pressure
*V*_*eed*_: effective LV end-diastolic volume.
